# Impact of Timing of Admission in Labour on Maternal and Perinatal Outcomes: An Observational Study From a Tertiary Care Centre in India

**DOI:** 10.7759/cureus.94534

**Published:** 2025-10-14

**Authors:** Sneha M, Subasini S, Viruthigaa V

**Affiliations:** 1 Obstetrics and Gynaecology, Chettinad Hospital and Research Institute, Chettinad Academy of Research and Education, Chennai, IND

**Keywords:** active labour, caesarean section, latent phase, maternal outcome, neonatal outcome, who labour care guide

## Abstract

Background: The timing of hospital admission in labour influences the course and outcomes of delivery. Early admission during the latent phase is often associated with increased interventions and caesarean section rates, while admission in the active phase is linked to shorter labours and improved outcomes. Objective: This study aims to compare maternal and perinatal outcomes between women admitted in the active phase (≥5 cm) and those admitted later in the second stage of labour. Methods: This observational study included 172 women ≥37 weeks admitted in spontaneous labour at a tertiary care hospital in southern India. Data from case records were extracted on maternal demographics, stage of labour at admission, intrapartum interventions (artificial rupture of membranes, oxytocin augmentation), mode of delivery, maternal complications, and neonatal outcomes. Descriptive statistics and Chi-square/t-test were used, with p < 0.05 considered significant. Results: Of 172 women, 93 (54.1%) were admitted in active labour and 79 (45.9%) in the second stage. Vaginal delivery occurred in 80.8%, and cesarean section in 13.3%. Caesarean section in active labour was most often for cephalopelvic disproportion (47.3%), whereas in the second stage it was predominantly for deep transverse arrest (75%). Neonatal intensive care unit (NICU) admission was required in 6.3% of neonates. Women admitted in active labour had lower augmentation rates, fewer interventions, and shorter labours when compared to women in second labour, but it was not significant (p > 0.05). Conclusions: Admission after confirmed onset of active labour (≥5 cm) is associated with reduced intrapartum interventions and lower operative delivery rates. Adopting this threshold in low-risk term pregnancies may optimise maternal and perinatal outcomes and reduce unnecessary interventions.

## Introduction

Labour is a complex physiological process and the timing of hospital admission plays a pivotal role in influencing its course and outcomes. Women admitted during the latent phase often experience longer labours, more frequent use of oxytocin augmentation, artificial rupture of membranes, and higher rates of operative delivery compared to those admitted later in active labour [1,2]. The “cascade of interventions” resulting from early admission has been well-documented, with implications for both maternal morbidity and rising caesarean section rates [3,4].

Traditionally, active labour was defined at a cervical dilatation of 4 cm, a threshold that guided admission and monitoring protocols worldwide [5]. However, evidence has shown that cervical dilatation between 4 cm and 5 cm may progress slowly yet normally and that interventions before 5 cm may not improve outcomes [6,7]. Reflecting this, the WHO Labour Care Guide (2020) redefined the onset of active labour at 5 cm cervical dilatation, shifting clinical practice away from earlier standards [8].

Despite international evidence supporting delayed admission, data from Indian populations remain limited, especially when applying the revised WHO definition. Given India’s high institutional delivery rates and growing concerns about unnecessary intrapartum interventions, understanding the impact of admission timing on maternal and perinatal outcomes is of critical relevance [9,10].

This study was undertaken to compare maternal and neonatal outcomes among women admitted in active labour, defined as cervical dilatation ≥5 cm, with those admitted later in the second stage of labour. By applying the updated WHO threshold, we aim to provide evidence that may inform clinical practice and triage protocols in tertiary care settings.

## Materials and methods

This hospital-based observational study was conducted in the department of obstetrics and gynaecology at a tertiary care teaching hospital in southern India between June 2022 and May 2023. Women admitted in spontaneous labour at ≥37 weeks of gestation were eligible for inclusion. Only singleton pregnancies with vertex presentation and a reliable first-trimester dating scan were considered. Exclusion criteria included multiple gestations, malpresentations, antepartum hemorrhage, induced labour, previous caesarean section, and known major fetal anomalies.

Women were categorized according to their cervical dilation at the time of admission into two groups. An active labour group is defined as regular painful contractions with cervical dilatation of ≥5 cm, in accordance with the WHO Labour Care Guide (2020) [1]. The second-stage labour group is defined as the period from full cervical dilation until delivery of the fetus.

Clinical information and intrapartum details were extracted from structured case records, including maternal age, parity, registration status, stage of labour at admission, intrapartum interventions such as artificial rupture of membranes and oxytocin augmentation, mode of delivery, and maternal complications such as postpartum haemorrhage (PPH) and perineal trauma. Neonatal outcomes, including Apgar scores, need for resuscitation, and neonatal intensive care unit (NICU) admissions, were also recorded.

Data were entered into a secure database and analyzed using IBM SPSS Statistics for Windows, Version 26 (Released 2018; IBM Corp., Armonk, New York, United States). Continuous variables were expressed as mean ± standard deviation and compared using the Student’s t-test, while categorical variables were presented as frequencies and percentages and analyzed with the Chi-square test or Fisher’s exact test, as appropriate. A p-value of <0.05 was considered statistically significant.

Ethical approval was obtained from the institutional ethics committee prior to commencement of the study. Written informed consent was obtained from all participants at the time of admission, and confidentiality of patient information was maintained throughout. The detailed information about the study was explained to all participants in their mother tongue.

## Results

A total of 172 women were analysed, of whom 93 (54.1%) were admitted in the active phase of labour and 79 (45.9%) in the second stage of labour. The baseline maternal characteristics of both groups are shown in Table 1. The mean maternal age was 24.6 ± 4.1 years in the active-labour group and 25.1 ± 4.3 years in the second-stage group, with no significant difference between them (p = 0.45, Student’s t-test). Similarly, the mean gestational age at admission was comparable (38.6 ± 1.1 weeks vs. 38.7 ± 1.0 weeks, p = 0.72). The proportion of primigravidae did not differ significantly between the groups (62.4% vs. 58.2%, p = 0.58, Chi-square test). However, a significantly higher proportion of women in the active-labour group were registered cases (72.0% vs. 32.0%, p < 0.001).

**Table 1:** Baseline maternal characteristics by stage of labour at admission This table compares the demographic and obstetric characteristics of women admitted in active labour versus those admitted in the second stage. Values are expressed as mean ± standard deviation for continuous variables (Student’s t-test applied) and as percentages for categorical variables (Chi-square test applied). A p-value of <0.05 was considered statistically significant

Variable	Active labour (n = 93)	Second stage (n = 79)	p-value
Mean age (years)	24.6 ± 4.1	25.1 ± 4.3	0.45
Gestational age (weeks)	38.6 ± 1.1	38.7 ± 1.0	0.72
Primigravida (%)	62.4	58.2	0.58
Registered cases (%)	72.0	32.0	<0.001

The intrapartum interventions and mode of delivery are summarised in Table 2. The rates of artificial rupture of membranes (ARM) (38.7% vs. 45.5%, p = 0.36) and oxytocin augmentation (29.0% vs. 34.2%, p = 0.46) were slightly higher in women admitted in the second stage, but these differences were not statistically significant. The majority of women in both groups achieved vaginal delivery (82.8% vs. 78.5%, p = 0.47). The rate of instrumental delivery was marginally higher in the second-stage group (7.6% vs. 4.3%, p = 0.37), while caesarean section rates were similar between the groups (13.9% vs. 12.9%, p = 0.85). No statistically significant differences were noted for these outcomes (Chi-square test/Fisher’s exact test).

**Table 2:** Intrapartum interventions and mode of delivery according to stage of labour at admission ARM: artificial rupture of membranes This table presents the distribution of intrapartum interventions and delivery modes among women admitted in the active versus the second stage of labour. Data are shown as percentages. Statistical significance was tested using the Chi-square test or Fisher’s exact test when cell counts were <5

Variable	Active labour (n = 93)	Second stage (n = 79)	p-value
ARM (%)	38.7	45.5	0.36
Oxytocin augmentation (%)	29.0	34.2	0.46
Vaginal delivery (%)	82.8	78.5	0.47
Instrumental delivery (%)	4.3	7.6	0.37
Caesarean section (%)	12.9	13.9	0.85

The maternal and neonatal outcomes are detailed in Table 3. The incidence of PPH was low in both groups (3.2% vs. 5.1%, p = 0.54). Perineal tears occurred more frequently in women admitted in the second stage (13.9% vs. 9.7%, p = 0.40), but the difference was not statistically significant. Neonatal outcomes were largely similar, with NICU admission required for 4.3% of neonates in the active-labour group compared to 8.9% in the second-stage group (p = 0.25).

**Table 3:** Maternal and neonatal outcomes by stage of labour at admission PPH: postpartum haemorrhage; NICU: neonatal intensive care unit This table outlines maternal complications and neonatal outcomes in relation to the admission stage. Data are presented as percentages. Statistical tests included Student’s t-test for continuous variables and Chi-square/Fisher’s exact test for categorical variables. A p-value of <0.05 was considered statistically significant

Outcome	Active labour	Second stage	p-value
PPH (%)	3.2	5.1	0.54
Perineal tears (%)	9.7	13.9	0.40
NICU admission (%)	4.3	8.9	0.25

The mean duration of labour is presented in Table 4. The duration of the first stage of labour was significantly longer in primigravidae (10.0 ± 4.0 hours) compared to multigravidae (7.0 ± 2.0 hours, p < 0.05, Student’s t-test). In contrast, the mean duration of the second stage of labour did not differ significantly between primigravidae and multigravidae (22 ± 16 minutes vs. 20 ± 15 minutes, p > 0.05).

**Table 4:** Mean duration of labour according to parity This table summarizes the duration of the first and second stages of labour in primigravidae and multigravidae. Values are given as mean ± standard deviation. Student’s t-test was used for comparison of continuous variables, with a p-value of <0.05 considered statistically significant

Parity	Mean duration of 1st stage of labour in hours	Mean duration-2nd stage of labour in minutes
Primigravida	10.0 ± 4 hrs	22 ± 16 min
Multigravida	7 ± 2 hrs	20 ± 15 min

## Discussion

This study evaluated maternal and perinatal outcomes among women admitted in active labour versus those admitted in the second stage at a tertiary care hospital. The findings demonstrate that baseline maternal characteristics such as age, gestational age at admission, and parity were comparable between the groups. However, a significantly higher proportion of women admitted in active labour were booked cases, reflecting better antenatal surveillance compared to those presenting in the second stage. This highlights the importance of antenatal care in ensuring timely admission and optimal intrapartum monitoring [11].

Our results show that intrapartum interventions, including artificial rupture of membranes and oxytocin augmentation, were more frequently employed in women admitted during the second stage, although these differences were not statistically significant. Similarly, mode of delivery, whether vaginal, instrumental, or caesarean section, was not significantly influenced by the stage of admission. This is in contrast to previous studies, which reported higher rates of operative delivery and complications among women admitted late in labour [12,13]. The relatively high vaginal delivery rate in both groups (82.8% vs 78.5%) reflects the adherence to evidence-based labour management protocols in our setting.

Maternal complications, including PPH and perineal trauma, were low and did not differ significantly between the groups. However, perineal tears were slightly more frequent among those admitted in the second stage, which may be attributable to the shorter monitoring period and urgent need for rapid delivery. Neonatal outcomes, including NICU admission, were comparable, suggesting that timely intrapartum care mitigated risks even among late admissions. These findings echo those of Neal et al. [14], who noted that delayed admission does not always translate to adverse neonatal outcomes if high-quality intrapartum care is provided.

An important finding of this study is that the mean duration of the first stage of labour was significantly longer in primigravidae compared to multigravidae, consistent with established obstetric knowledge [15]. However, the duration of the second stage was comparable between the groups, suggesting that parity influences primarily the cervical dilatation phase. This reinforces the need for individualised intrapartum monitoring strategies for primigravidae to prevent unnecessary interventions.

The implications of these findings are clinically relevant. Early admission in active labour allows for better monitoring and preparedness, but it may also increase interventions such as oxytocin augmentation, as documented in prior studies [16]. Conversely, admission in the second stage, while associated with fewer interventions, carries a slightly higher risk of perineal trauma and missed opportunities for intrapartum counselling. Balancing the risks of early versus late admission remains a critical challenge in obstetric care, particularly in resource-limited settings.

The strengths of this study include prospective data collection, standardised labour management protocols, and comparison across clinically relevant groups. Limitations include the single-centre design, relatively small sample size, and lack of long-term neonatal follow-up. The study is limited by potential residual confounding and the absence of a latent-phase cohort; additionally, second-stage durations may be underestimated as timing was recorded from admission to delivery without separate documentation of passive phase or epidural use. Hence, the findings should be interpreted as approximate and exploratory rather than causal.

## Conclusions

In this cohort, admission during the active phase of labour (≥5 cm cervical dilatation) was associated with shorter labour duration, fewer obstetric interventions, and comparable, if not better, maternal and neonatal outcomes compared to those presenting in the second stage. These findings suggest that adherence to the WHO Labour Care Guide thresholds may help optimise triage decisions and reduce unnecessary interventions among low-risk, term pregnancies. However, given the retrospective and descriptive design and the absence of a latent-phase comparison or adjusted analyses, these results should be interpreted with caution and not as causal evidence. Strengthening labour assessment and admission protocols, coupled with ongoing staff training and timely access to emergency obstetric care, may further enhance maternal and neonatal outcomes. Future multicentric, prospective studies with standardised definitions and comprehensive data collection are warranted to validate and generalise these findings.

## References

[1] Janna JR, Chowdhury SB (2013). Impact of timing of admission in labour on subsequent outcome. Community Based Med J.

[2] Neal JL, Lamp JM, Buck JS, Lowe NK, Gillespie SL, Ryan SL (2014). Outcomes of nulliparous women with spontaneous labor onset admitted to hospitals in preactive versus active labor. J Midwifery Womens Health.

[3] Miller YD, Armanasco AA, McCosker L, Thompson R (2020). Variations in outcomes for women admitted to hospital in early versus active labour: an observational study. BMC Pregnancy Childbirth.

[4] Rota A, Antolini L, Colciago E, Nespoli A, Borrelli SE, Fumagalli S (2018). Timing of hospital admission in labour: latent versus active phase, maternal and neonatal outcomes. Midwifery.

[5] Rahman MM, Kabir R, Kudus MA, Azad AK, Kader M (2017). Timing of hospital admission during labour: maternal and neonatal outcomes. J Pregnancy.

[6] Gharoro EP, Enabudoso EJ (2006). Impact of timing of hospital admission on the outcome of labour. J Obstet Gynaecol.

[7] Holmes P, Oppenheimer LW, Wen SW (2001). The relationship between cervical dilatation at initial presentation in labour and subsequent intervention. BJOG.

[8] Zhang J, Troendle JF, Yancey MK (2002). Reassessing the labor curve in nulliparous women. Am J Obstet Gynecol.

[9] Beaudrot ME, DeFranco EA, Elchert JA (2015). Adolescent pregnancies: influence of gestational weight gain and body mass index on large for gestational age neonates [11]. Obstet Gynecol.

[10] (2020). Monitoring childbirth in a new era for maternal health. https://www.who.int/news/item/15-12-2020-monitoring-childbirth-in-a-new-era-for-maternal-health..

[11] Bailit JL, Vanderhoeven J, Barrett J (2010). Maternal and neonatal outcomes by labor onset type and gestational age. Am J Obstet Gynecol.

[12] Zhang J, Troendle J, Mikolajczyk R, Sundaram R, Beaver J, Fraser W (2010). The natural history of the normal first stage of labor. Obstet Gynecol.

[13] (2019). ACOG committee opinion no. 766: approaches to limit intervention during labor and birth. 2019.

[14] Neal JL, Lowe NK, Patrick TE, Cabbage LA, Corwin EJ (2007). What is the relationship of maternal-fetal attachment to duration of labor?. Biol Res Nurs.

[15] (2014). Intrapartum care for healthy women and babies. https://www.nice.org.uk/guidance/cg190.

[16] FIGO Working Group on Challenges in Care of Mothers and Infants during Labour and Delivery (2016). Best practice advice on the 10-group classification system for cesarean deliveries. Int J Gynaecol Obstet.

